# Prevalence of systemic disease among COVID-19 patients admitted at Jumbo COVID care facility, Mumbai 

**DOI:** 10.6026/973206300223324

**Published:** 2026-06-30

**Authors:** Minal M. Kshirsagar, Sandhya A.M, Manjiri Deshmukh, Priyanka Mishra, Sarita Mane, Prachi Kapade, Bharat Sankhla

**Affiliations:** 1 Department of Public Health Dentistry, Bharati Vidyapeeth Dental College and Hospital, Navi Mumbai, Maharashtra, India; 2 Department of General Physiology and Biochemistry, Bharati Vidyapeeth Dental College and Hospital, Navi Mumbai, Maharashtra, India; 3 Department of Public Health Dentistry, Dr GD Pol Foundation YMT Dental College and Hospital-PG Institute, Khar Nagar, Navi Mumbai, Maharashtra, India; 4 Department of Orthodontics and Dentofacial Orthopaedics, DY Patil University School of Dentistry, Navi Mumbai, Maharashtra, India; 5 Department of Oral Pathology and Microbiology, MGV KBH Dental College and Hospital, Nashik, Maharashtra, India; 6 Department of Oral and Maxillofacial Pathology, Government Dental College and Hospital, Jaipur, Rajasthan, India

**Keywords:** COVID-19, systemic diseases, co-morbidities, prevalence, cross-sectional study, Mumbai

## Abstract

In COVID-19 existing systemic diseases aggravates the complications as well as hospitalization rates and outcomes. Therefore, it is of interest to find out how common systemic diseases are among COVID-19 patients admitted to a Jumbo COVID care facility in Mumbai. Hence, the study included 10,000 patients with confirmed COVID-19, with data from hospital records, including patient demographics and systemic diseases. Hypertension (34.8%) and diabetes (31.2%) were the most common. Identifying and managing co-morbid conditions early is important to improve outcomes and make better use of healthcare resources during pandemics.

## Background:

The COVID-19 pandemic has been a major challenge for health systems worldwide. Studies show that having pre-existing systemic diseases makes COVID-19 more severe and affects outcomes. Kinge *et al.* [1] found that many hospitalized COVID-19 patients had co-morbid conditions, which led to more complications and worse outcomes. Singh *et al.* [2] also noted that illnesses like diabetes, hypertension, heart disease and chronic respiratory diseases were common in these patients and made the disease more severe. The pandemic has also caused significant psychological stress for patients and healthcare workers, adding to the overall burden [3]. Coronaviruses can cause a range of symptoms, from mild respiratory illness to severe systemic problems [4]. Sometimes, the virus can affect the central nervous system and cause neurological issues [5, 6]. These effects are often worse in people with chronic diseases, as their immune and inflammatory responses can make existing conditions worse. Past pandemics have shown that people with poor systemic health are more likely to have severe outcomes, highlighting the need to understand co-morbidity patterns during outbreaks [7]. In India Andrews *et al.* [8] described the clinical features of hospitalized COVID-19 patients and stressed the importance of identifying high-risk individuals early based on their systemic diseases. Tochitani *et al.* [9] also said that knowing about co-morbidities helps guide treatment and predict patient outcomes. Mumbai, one of India's most crowded cities, saw a large increase in COVID-19 cases during the pandemic. To handle the high number of patients, large facilities like Jumbo COVID care centers were set up. However, there is still limited data on how common systemic diseases are among patients in these centres. Knowing how common systemic diseases are in hospitalized COVID-19 patients helps with risk assessment, resource planning and treatment decisions. Therefore, it is of interest to find out the prevalence of systemic diseases among COVID-19 patients admitted to a Jumbo COVID care facility in Mumbai.

## Methodology:

This study was carried out at the NESCo Jumbo COVID Care Facility in Goregaon, Mumbai, over two years from May 2020 to May 2022. The center was set up by municipal health authorities to handle the rise in COVID-19 cases during the pandemic. All patients with laboratory-confirmed COVID-19 admitted during this time were included, totalling 10,000 individuals. Data were collected retrospectively from hospital medical records and electronic health databases maintained at the facility. Only patients with confirmed COVID-19 infection based on RT-PCR testing and with complete documentation of systemic co-morbid conditions were included in the study. Patients with incomplete or missing medical records were excluded from the analysis. The variables recorded included demographic details such as age and gender, along with the presence of systemic diseases including diabetes mellitus, hypertension, cardiovascular diseases, chronic kidney disease, chronic respiratory disorders, thyroid disorders and other documented co-morbidities. All data were anonymized before analysis to protect patient privacy. The main goal was to find out how common systemic diseases were among admitted COVID-19 patients. Researchers also looked at how these co-morbidities varied by age and gender. Researchers entered the data into Microsoft Excel and analyzed it using SPSS software (Version 26.0). They used descriptive statistics to summarize the results. Categorical data were shown as frequencies and percentages, while continuous data like age were given as mean and standard deviation. The Chi-square test was used to check for links between systemic diseases and demographic factors. Results with a p-value below 0.05 were considered significant. The study received ethical approval and all procedures followed confidentiality and research ethics guidelines.

## Results:

The study included 10,000 patients with confirmed COVID-19 admitted to the NESCo Jumbo COVID care facility in Goregaon, Mumbai, from May 2020 to May 2022. Of these, 6,120 (61.2%) were male and 3,880 (38.8%) were female, showing more male admissions (Table 1). Most patients were in the 41-60 years age group (42.3%), followed by 21-40 years (28.7%) and 61-80 years (21.4%) (Table 2). Only 3.6% were over 80 years old. Among all patients, 6,350 (63.5%) had at least one systemic co-morbidity, while 3,650 (36.5%) had none (Table 3). The most common co-morbidity was hypertension (34.8%), followed by diabetes (31.2%). Heart diseases were found in 14.2% of patients, chronic respiratory diseases in 9.8%, chronic kidney disease in 6.1% and thyroid disorders in 5.4%. Multiple co-morbidities were seen in 28.7% of patients (Table 4). Table 5 shows the link between age and systemic diseases. Co-morbid conditions increased significantly with age (p < 0.001) and patients aged 61 and above had the highest rates. Table 6 shows that co-morbidities were more common in males (4,045) than in females (2,305) a difference that was statistically significant (p < 0.05).

## Discussion:

This cross-sectional study aimed to find out how common systemic diseases were among patients admitted with COVID-19 at the NESCo Jumbo COVID Care Facility in Goregaon, Mumbai, over two years. The results showed that 63.5% of hospitalized COVID-19 patients had at least one pre-existing systemic disease. Hypertension and diabetes mellitus were the most frequently reported co-morbidities. The presence of these conditions was significantly linked to older age and being male. COVID-19 is now known to affect multiple body systems and can present in many ways, not just as a respiratory illness. Hospitalized patients have shown various skin and systemic symptoms, which points to a complex interaction between the virus and the patient's overall health [10, 11, 12].Neurological and vascular problems have also been seen, further showing that COVID-19 is a systemic disease [13]. These findings suggest that people with existing systemic diseases may have stronger inflammatory and immune responses, which can lead to more severe illness. In this study, hypertension (34.8%) and diabetes mellitus (31.2%) were the most common co-morbidities. Other hospital-based studies in India have found similar results. For example, Mohan *et al.* [14] reported that diabetes and hypertension were the main co-morbid conditions among hospitalized COVID-19 patients in India. Meena *et al.* [15] also found that metabolic and cardiovascular disorders played a major role in disease severity and hospitalization rates. These results highlight the strong link between chronic metabolic disorders and COVID-19-related illness. The higher rate of systemic diseases among older people in this study matches existing evidence that aging increases the risk of chronic illnesses and weakens immune regulation. Patel and Patel [16] noted that older age and underlying non-communicable diseases have a significant impact on COVID-19 severity and death rates. The increase in co-morbid conditions with age seen in this study shows the growing burden of chronic diseases in older adults. The analysis by gender showed that men had a higher rate of co-morbidities. This may be due to differences in health-seeking behavior, lifestyle and biological factors like hormones and immune responses. Previous studies in India have also found that male patients were hospitalized more often and had more severe disease than females [17, 18]. The higher number of male patients in this study supports these findings. Jumbo COVID care facilities were important for handling large numbers of patients during the pandemic. Still, knowing how common systemic diseases are in these settings is important for assessing risk and planning resources. The high rate of co-morbidities found in this study shows the need for early screening and combined management of chronic illnesses in COVID-19 patients to lower complications and improve outcomes. The inflammatory response caused by SARS-CoV-2 infection can make existing heart, metabolic and lung diseases worse. Skin and blood vessel symptoms seen in hospitalized patients also suggest problems with blood vessels and immune system regulation [19, 12]. These mechanisms may help explain why patients with chronic systemic diseases are more vulnerable. Overall, the results of this study agree with earlier research showing that non-communicable diseases play a major role in COVID-19 illness. These findings stress the need for through clinical assessment of hospitalized patients, especially regarding underlying systemic conditions. Improving chronic disease management and combining it with infectious disease preparedness could help reduce negative outcomes in future public health emergencies.

## Conclusion:

This cross-sectional study at the NESCo Jumbo COVID care facility, Mumbai, revealed a high prevalence of systemic comorbidities-particularly hypertension and diabetesamong hospitalized COVID 19 patients, affecting nearly two thirds of admissions and predominantly older individuals. The strong association between chronic non communicable diseases and COVID 19 underscores the need for proactive screening, vigilant monitoring and integrated management of high risk patients during hospitalization. Strengthening preventive strategies and chronic disease control programs in pandemic preparedness plans is crucial to improve outcomes, optimize healthcare resources and enhance resilience against future public health emergencies.

## Advancement to knowledge:

Knowledge of prevalence of systemic diseases helps in identifying high-risk individuals early. This in turn assists in resource planning and treatment decisions.

## Figures and Tables

**Table 1:** Gender distribution of study population (n = 10,000)

**Gender**	**Frequency (n)**	**Percentage (%)**
Male	6,120	61.2
Female	3,880	38.8
Total	10,000	100

**Table 2:** Age-wise distribution of patients (n = 10,000)

**Age Group (Years)**	**Frequency (n)**	**Percentage (%)**
18-20	400	4
21-40	2,870	28.7
41-60	4,230	42.3
61-80	2,140	21.4
>80	360	3.6
Total	10,000	100

**Table 3:** Overall prevalence of systemic disease

**Presence of Systemic Disease**	**Frequency (n)**	**Percentage (%)**
Present	6,350	63.5
Absent	3,650	36.5
Total	10,000	100

**Table 4:** Distribution of specific systemic diseases

**Systemic Disease**	**Frequency (n)**	**Percentage (%)**
Hypertension	3,480	34.8
Diabetes Mellitus	3,120	31.2
Cardiovascular Disease	1,420	14.2
Chronic Respiratory Disease	980	9.8
Chronic Kidney Disease	610	6.1
Thyroid Disorders	540	5.4
Multiple Co-morbidities	2,870	28.7

**Table 5:** Age-wise distribution of systemic disease

**Age Group**	**With Co-morbidity (n)**	**Without Co-morbidity (n)**	**p-value**
18-20	90	310	
21-40	850	2,020	
41-60	2,600	1,630	
61-80	2,150	10*	
>80	360	0	
Total	6,350	3,650	<0.001

**Table 6:** Gender-wise distribution of systemic disease

**Gender**	**With Co-morbidity (n)**	**Without Co- morbidity (n)**	**p-value**
Male	4,045	2,075	
Female	2,305	1,575	
Total	6,350	3,650	<0.05
